# Immunotherapies for Breast Cancer: From Checkpoint Inhibition to Emerging Cellular Therapies

**DOI:** 10.3390/cancers18060911

**Published:** 2026-03-11

**Authors:** Ismini Tsagkaraki, Isaac Gannon, Alexandros Rampotas, Devika Singh, Harriet Roddy, Diego Ottaviani, Claire Roddie

**Affiliations:** 1 Oncology Department, Royal Berkshire NHS Foundation Trust, Reading RG1 5AN, UK; 2 Haematology Department, Cancer Institute, University College London, London WC1E 6DD, UK; 3 Haematology Department, University College London Hospitals NHS Foundation Trust, London NW1 2BU, UK; 4 Oncology Department, University College London Hospitals NHS Foundation Trust, London NW1 2BU, UK

**Keywords:** CAR-T, cellular therapies, breast cancer, immunotherapy

## Abstract

Breast cancer remains a leading cause of cancer-related morbidity and mortality worldwide, with therapeutic response being shaped by the unique biology of each breast cancer subtype. Immunotherapy has emerged as a transformative approach in selected disease subtypes, with the most successful results being found in relation to triple negative breast cancer (TNBC). Immune checkpoint inhibitors (ICIs) have transformed the management of many solid tumours. In breast cancer, they have demonstrated clinical benefit in TNBC when combined with chemotherapy, establishing a new standard of care in both early-stage and metastatic settings. However, the majority of breast cancers exhibit intrinsic or acquired resistance to checkpoint blockade, driven by low tumour immunogenicity and an immunosuppressive tumour microenvironment. Recent advances in cellular immunotherapy could represent the next frontier in the therapeutic landscape of breast cancer. Chimeric antigen receptor (CAR) T cell targeting antigens such as HER2, ROR1, MUC1, mesothelin, and B7-H3 are entering early-phase clinical evaluation with results eagerly awaited. Parallel approaches, including tumour-infiltrating lymphocyte (TIL) therapy, T cell receptor (TCR)-engineered T cells, and CAR-natural killer (CAR-NK) platforms, offer alternative mechanisms to overcome antigen presentation barriers and immune evasion. This review summarises current clinical evidence for immunotherapies in breast cancer, highlights emerging cellular strategies, and discusses key challenges including antigen specificity, off-tumour toxicity, and tumour microenvironment-mediated resistance. Future progress will likely depend on rational combination approaches and next-generation engineered immune cell platforms to achieve durable and personalised clinical benefit.

## Simple Summary

Immunotherapy has been demonstrated to be clinically beneficial in breast cancer, particularly in subsets such as triple-negative disease. These advances provide a strong rationale for the development of cellular therapies, including CAR-T cells, to overcome the limitations of current treatments and improve outcomes in difficult-to-treat tumours. However, major challenges remain, including the identification of truly tumour-specific antigens and the immunosuppressive tumour microenvironment, both of which pose significant barriers to safety and efficacy.

## 1. Introduction

Breast cancer is the most common type of cancer in the United Kingdom (UK). In total, 1 in 7 women will be diagnosed with breast cancer in their lifetime; men can also be affected, accounting for less than 1% of all cases. It is the second most common cause of cancer death in the UK.

Risk factors for breast cancer include age and genetics, in addition to environmental and lifestyle factors such as alcohol, combined oral contraceptives and radiation. By mitigating or eliminating these factors, it is estimated that around 23% of UK cases may be preventable [1].

Breast cancer typically arises from mutated cells in the lobules or ducts within the breast tissue that divide uncontrollably. Symptoms may be absent in small cancers and therefore mammography-based screening is essential for early detection, while AI-assisted tools for screening mammograms are increasingly getting better at identifying even small lesions [2]. However, the challenges of pre-emptive cancer population screening remain: false positives can lead to unnecessary procedures, and although the system is designed to minimise false negatives, they still occur [3]. Evidence suggests that screening can reduce mortality, but this is likely to a lesser extent than most healthcare systems anticipate [4].

Breast cancer is a highly heterogenous malignancy and based on the molecular expression of the oestrogen receptor (ER), progesterone receptor (PR), human epidermal growth factor receptor 2 (HER-2) and Ki-67 (proliferation) status; it can be classified into four subtypes: Luminal A (HER-2-negative, ER-positive, PR-positive, low proliferation), Luminal B (HER-2-negative, ER-positive, PR-positive, high proliferation), HER-2 amplified (HER-2-positive and ER-positive or PR-positive or both ER- and PR-positive OR HER-2-positive and ER- and PR-negative), and triple negative breast cancer (TNBC) (HER-2-negative, ER-negative, PR-negative) [5]. There are further efforts to more accurately molecularly classify HER-2-positive breast cancer [6].

Treatment strategies are determined by the molecular subtype and clinical stage of the disease. In early breast cancer, the treatment intent is curative, aiming at eradication of the tumour and prevention of disease recurrence. Surgery is used for tumour eradication whereas radiotherapy is administered postoperatively as adjuvant treatment to prevent local recurrence. Systemic therapies such as endocrine therapy, chemotherapy and immunotherapy can be used in the neoadjuvant (preoperative) and/or adjuvant (postoperative) settings for downstaging and prevention of local and distal recurrence [7].

For metastatic disease, systemic treatment together with local therapies (excision and radiotherapy) are used with the aim of symptom control and life prolongation. Systemic treatment selection is based on the molecular subtype of cancer. Luminal A and B subtypes are generally responsive to endocrine and targeted treatments including CDK4/6 inhibitors, whereas HER-2 amplified breast cancer responds effectively to anti-HER-2 therapies [8]. In TNBC, the lack of specific targets, chemotherapy resistance and the aggressive clinical course remain the main therapeutic challenges. For this reason novel, effective, targeted combination treatments are needed for optimal management of TNBC [9].

Immunotherapy is one of the most remarkable breakthroughs in cancer treatment in the last 20 years. Immune checkpoint inhibitors (ICIs) allow the host immune system to fight and destroy malignant cells, including some subtypes of breast cancer. By targeting immune checkpoint proteins such as programmed-death receptor (PD-1) on T cells, B-cells, and antigen-presenting cells, and PD ligand (PD-L1) on tumour cells and cytotoxic T-lymphocyte-associated protein 4 (CTLA 4) on T cells, it is possible to overcome checkpoint-mediated immune inhibitory signals exploited by cancer cells to evade the immune response [10], while dendritic cell PD-1 inhibition may further enhance the response. Examples include the PD-L1 inhibitors atezolizumab [11], avelumab and durvulumab, the PD-1 inhibitors pembrolizumab and nivolumab, and the CTLA-4 inhibitor ipilimumab. These have been used with great clinical success in melanoma [12], renal cell carcinoma [13] and non-small cell lung cancer (NSCLC) [14], establishing immunotherapy as a new standard of care in these cancer types.

In contrast to the exemplar tumours above, breast cancer presents a major challenge to ICI therapy due to the immunosuppressive nature of the breast tumour itself and its surrounding tumour microenvironment (TME). Firstly, breast cancer cells exploit critical signalling pathways to escape the immune system [15]. Secondly, they typically downregulate MHC class I gene expression which, together with impaired antigen processing, reduces antigen presentation (and recognition by) CD8+ T cells. Other immune evasion mechanisms include physical (stromal) barriers [16], metabolic competition [17] and soluble immunosuppressive factors (e.g., TGF-β) [18], alongside recruitment of immunosuppressive regulatory T cells (Tregs), myeloid-derived suppressor cells (MDSCs) and tumour-associated macrophages (TAMs) [19]. Furthermore, although tumour-associated antigens (TAAs) such as HER-2, Muc-1 and CEA can be overexpressed, they usually lead to immune tolerance, while additional mutations such as TP53 and PIK can drive immune evasion and T cell exhaustion [20].

**Figure 1:**
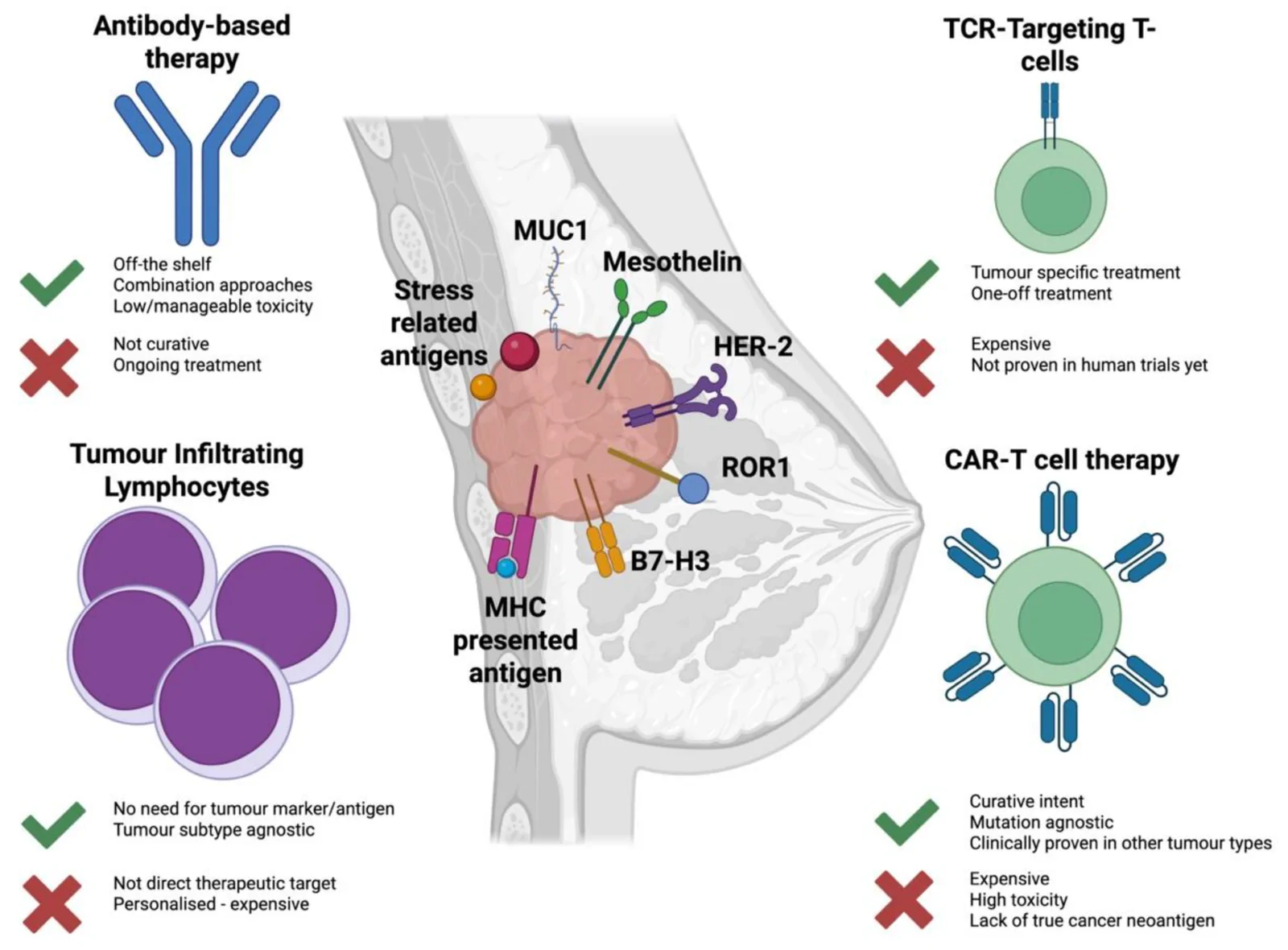
Different immunotherapeutic approaches targeting breast cancer. Antibody-based therapies, Tumour Infiltrating Lymphocytes, TCR-Targeting T-cells and CAR-T cell therapies are highlighted here. Advantages and disadvantages of each modality are highlighted with either a green check mark or a red cross respectively. In the middle, the most promising antigenic targets for TCR and CAR-T cell therapies are presented.

However, despite the biological challenges highlighted above, recent research developments have shown that immunotherapy has an important role to play in breast cancer management. Other than checkpoints inhibitors, this includes monoclonal-based therapies, tumour-infiltrating lymphocyte therapies, and TCR-based and CAR-T cellular therapies (Figure 1). Here, we present an overview of the latest developments in immunotherapy for breast cancer, with a focus on cellular therapies.

## 2. Current Immunotherapies in Breast Cancer

### 2.1. Immune Checkpoint Inhibitors (ICIs)

Immune checkpoint inhibitors (ICIs) have not secured regulatory approval for hormone receptor-positive (ER/PR+) or HER2-positive breast cancer outside of specific investigational settings. Although clinical trials have suggested potential activity [21], these approaches have not demonstrated sufficient efficacy to displace established standards of care, namely endocrine therapy in ER/PR+ disease and HER2-directed targeted therapies in HER2-positive tumours [11]. ICIs have been approved by the FDA and EMA for early and metastatic TNBC for specific patient groups, including the use of PD-L1 blockade [11].

The phase II trial KEYNOTE-086 and the subsequent phase III trial KEYNOTE-119 that were designed to evaluate pembrolizumab monotherapy in metastatic TNBC were negative for any improvement in progression-free or overall survival [22]. Despite the poor results of ICI monotherapy for breast cancer, it has been shown that chemotherapy in combination with ICIs can induce the release of tumour-associated neo-antigens and can induce immune surveillance [23]. These results led to the first approvals of ICI (in combination with chemotherapy) in breast cancer treatment [24]. The first ICI approved by the FDA for breast cancer was atezolizumab [25]. This PD-L1 inhibitor received accelerated approval based on the results of the IMpassion130 trial which demonstrated a PFS and OS improvement in an exploratory analysis when atezolizumab was combined with nab-paclitaxel compared with nab-paclitaxel alone in patients with previously untreated PD-L1 immune cell-positive metastatic triple-negative breast cancer [12]. However, the FDA later withdrew the approval as the IMpassion131 trial failed to show superior PFS or OS when atezolizumab was combined with paclitaxel. The European Medicines Agency (EMA) maintains approval of atezolizumab in combination with nab-paclitaxel for this indication [26].

The PD-1 inhibitor pembrolizumab is FDA- and EMA-approved for TNBC in the neoadjuvant and metastatic settings in combination with chemotherapy [27]. The approval was based on the phase III KEYNOTE-522 trial that demonstrated improved pathological complete response (pCR) and event-free survival (EFS) in early TNBC. In the United Kingdom, NICE has also approved pembrolizumab in high-risk early stage TNBC or locally advanced TNBC in the neoadjuvant setting in combination with chemotherapy, and as monotherapy in the adjuvant setting following surgery. In locally recurrent unresectable or metastatic TNBC, NICE has approved pembrolizumab with paclitaxel or nab-paclitaxel for patients who have not received chemotherapy for metastatic disease [28]. The KEYNOTE-355 trial also showed a median OS benefit of about 7 months in metastatic TNBC with PD-L1 CPS ≥ 10 when pembrolizumab was added to chemotherapy [29].

Trials designed to evaluate the efficacy of immunotherapy combinations in TNBC are ongoing, such as trials of dual checkpoint blockade alongside chemotherapy. Further, TKIs and antibody–drug conjugates (ADCs) are also under investigation. Ongoing and completed trials are summarised in Table 1, while more comprehensive assessment of ICIs has been performed by Debien et al. [30] and Villacampa et al. [31].

### 2.2. HER2-Targeted Therapies

Anti-HER2 monoclonal antibodies such as trastuzumab and pertuzumab have been shown to have an important role in the management of HER-2-positive breast cancers. Although the FDA labels them as targeted therapies, they have immune-mediated effects. They primarily block the HER2 receptor directly, inhibiting signalling and proliferation of cancer cells. They also work as ‘passive immunotherapy’ by activating NK cells via Fc receptors to kill HER-2-positive cancer cells. This is called antibody-dependent cellular cytotoxicity (ADCC). Antibody–drug conjugates (ADCs) can deliver a cytotoxic drug payload targeted to a specific tumour-associated antigen by using antibody specificity to direct the drug selectively into the cancer cells. HER-2-directed antibody–drug conjugates such as Trastuzumab Deruxtecan (T-DXd) and Trastuzmab Emtansine (T-DM1) have been approved for HER-2-positive metastatic breast cancers [32]. There are also several studies that evaluate immune checkpoint inhibitors in early and metastatic HER2-positive breast cancer [25].

However, big questions about the use of immunotherapy still remain. Defining up-front which patients will respond to ICI therapy is an ongoing research priority. Patients with high tumour mutational burden (TMB) may benefit from immunotherapy. High TMB indicates that the tumour is enriched with mutated antigens that have the potential to be recognised by endogenous T cells which could lead to immunological tumour rejection via a cytotoxic immune response [33]. Hormone-driven breast cancers generally possess low TMB and are less likely to respond to immunotherapy. On the other hand, TNBC and HER-2-positive breast cancers have a high TMB and are potentially more likely to respond to immunotherapy. The presence of tumour-infiltrating lymphocytes (TILs) such as CD4+ T cells, CD8+ T cells, Th1 CD4+ T cells and NK cells has also been suggested in clinical trials as a biomarker for prediction of response to ICIs [34,35]. Similarly, there are negative biomarkers such as CD4+ TRegs, TAMs, MDSc, B2M, HLA-A deletions [36] and JAK1/2 mutations [37] that potentially reduce the likelihood of tumour response to ICIs.

## 3. Emerging Cellular Therapies in Breast Cancer

### 3.1. CAR-T Cell Therapies

Exploiting tumour-associated surface antigens as therapeutic targets offers a promising avenue for MHC-independent T cell therapies. Chimeric antigen receptors (CARs) are engineered fusion proteins in which an antibody-derived single-chain variable fragment (scFv) is linked to the CD3ζ signalling domain of the TCR complex, enabling the bypass of native antigen presentation and redirecting T cell cytotoxicity toward tumour cells [38].

Subsequent “second-generation” CARs incorporated signalling domains from co-stimulatory receptors such as CD28 or 4-1BB, markedly enhancing proliferation, persistence, and antitumour function [39].

At present, seven CAR-T products have received FDA approval, all directed against B cell malignancies (STN: BL 125646/0, STN: BL 125643/0, STN: BL 125703/0, STN: BL 125714/0, STN: BL 125736/0, STN: BL 125746/0, STN: BL 125813/0).

By contrast, no CAR-T therapies are yet approved for breast cancer [40], but several products are currently undergoing early clinical evaluation (Table 2).

#### 3.1.1. HER2 Targeting CAR-T

The HER2 receptor is a cell-surface tyrosine kinase that drives tumour proliferation and survival when overexpressed, and its accessible extracellular domain combined with its oncogenic dependency makes it an attractive, though safety-challenging, target for CAR-T therapy.

HER2-specific CAR-T therapies remain in early clinical development. For instance, trial NCT03696030 is currently active and evaluating the safety of a second-generation HER2-CAR treatment for patients with brain or leptomeningeal metastases from HER2+ cancers. Another ongoing study, NCT04995003, is recruiting patients with HER2-positive solid tumours (including breast cancer) to test a second-generation HER2-CAR in combination with an immune checkpoint inhibitor. A bispecific CAR-T targeting both HER2 and the breast cancer-associated antigen TRAIL-R2 is also in pre-recruitment for a phase I trial (NCT06251544) in metastatic breast cancer.

To address the challenge of on-target, off-tumour toxicity, a phase I trial (NCT04650451) assessed GoCAR-T, a first-generation HER2-CAR incorporating a synthetic receptor that provides co-stimulation in the presence of rimiducid, a small molecule dimeriser drug that can induce the expression of the CAR co-stimulatory endodomain. However, this trial was suspended following a “dose-limiting toxicity” event reported in a sister trial using the same technology in an anti-PSCA CAR-T for prostate cancer [41]. Although it is difficult to fully disentangle the contributions to toxicity from the CAR construct versus the rimiducid-dependent receptor, HER2 likely presents a comparable, if not higher, risk of off-tumour toxicity [42]. While data from ongoing trials are needed to accurately assess the therapeutic potential of HER2-CAR-T cells, other groups are exploring strategies to mitigate off-tumour effects—for example, employing low-affinity HER2 antibodies as CAR binders to preferentially target tumour cells with high antigen expression [43].

#### 3.1.2. ROR1 Targeting CAR-T

Receptor tyrosine kinase-like orphan receptor 1 (ROR1) is a cell-surface receptor normally expressed only during embryonic development but re-expressed in aggressive breast cancers. It has been associated with tumour growth and metastasis while being largely absent from normal adult tissues [44]. A phase I trial in TNBC and NSCLC (NCT05274451), completed in 2025, evaluated anti-ROR1 CAR-T cells with enforced C-Jun expression, manufactured using proprietary protocols designed to limit T cell differentiation. Biopsy analysis showed evidence of CAR-T cell tumour infiltration and lysis, while peripheral blood monitoring revealed CAR-T expansion that correlated with the initial administered dose [45]. However, the best overall response rate was two PRs (in 16 patients); 1 trial patient died of pneumonitis, which was possibly associated with the CAR-T product, and so consequently the product is currently not being further pursued. Another, now terminated, phase I trial (NCT02706392) evaluated anti-ROR1 CARs in TNBC, NSCLC, and CLL. The therapy was generally well tolerated, with only one in ten TNBC patients experiencing grade 1 ICANS as the sole adverse event. However, efficacy was limited: most patients initially achieved stable disease but progressed within six months of treatment [46].

#### 3.1.3. MUC1 Targeting CAR-T

Mucin-1 (MUC1) is a transmembrane mucin protein that is overexpressed and abnormally glycosylated in many breast cancers promoting growth and immune evasion. It represents a good CAR-T target as its tumour-specific form is present on the surface of cancer cells at high density, with more restricted expression on normal tissues and only a minimal expression on activated T cells, B-cells and dendritic cells [47]. MUC1-directed CAR-T therapy remains in the early stages of clinical development, although current trials are beginning to incorporate additional T cell engineering strategies. NCT05812326 was a phase I 3 + 3 dose-escalation trial evaluating the safety of an anti-MUC1 CAR utilising PD-1 knockout T cells. Results demonstrated acceptable safety and tolerability: among 12 treated patients, no cytokine release syndrome (CRS) events above grade 3 were reported, and 5 patients achieved stable disease (SD), suggesting early signs of potential efficacy [48].

Another MUC1-focused study, NCT04020575, is an ongoing multi-arm trial testing a MUC1-C binder with two CAR architectures—a conventional 4-1BBζ CAR and a CD28ζ CAR incorporating point mutations in ITAMs 2 and 3 of the CD3ζ endodomain to enhance T cell persistence [49]. Preliminary data from the 4-1BBζ CAR cohort (8 patients) indicate a favourable safety profile, with no observed off-target toxicity. Three patients experienced grade 3 or lower CRS, while one patient developed a grade 5 serious adverse event (SAE) [50].

#### 3.1.4. Mesothelin Targeting CAR-T

Mesothelin is a cell-surface glycoprotein normally expressed at low levels on mesothelial cells but overexpressed in aggressive breast cancers, particularly triple-negative disease [51]. There are currently two reported trials of known status investigating mesothelin-specific CAR-T therapy. NCT02792114 an active phase I trial evaluating the safety of the product; however, no data have yet been reported from this study. NCT02414269 is an active phase I/II trial for malignant pleural diseases, including metastatic lung and breast cancers as well as malignant pleural mesothelioma. Among the 27 patients initially treated—some of whom also received PD-1 blocking antibodies—no CRS or neurotoxicity events of grade 2 or higher were observed. Stable disease (SD) lasting more than six months was reported in eight patients, and two patients achieved complete metabolic responses as assessed by PET imaging [52]. While these results are encouraging, additional data from higher-dose and BC specific cohorts in this ongoing trial will be needed to confirm efficacy.

#### 3.1.5. B7-H3 Targeting CAR-T

Of the CAR-T targets described previously, B7-H3-targeting CARs are at the earliest stage of clinical development for breast cancer. B7-H3 is highly expressed in breast cancer cells inhibiting T cell responses, with very little to no expression in normal adult tissues, including immune cells [53]. Two trials are currently recruiting patients with advanced lung cancer and TNBC (NCT05341492) and relapsed/refractory TNBC (NCT06347068). Encouragingly, early phase I data of B7-H3 CARs in other solid tumour indications (NCT04185038, NCT05241392) have demonstrated minimal toxicity, even in the context of repeated dosing, with some signals for potential efficacy [54,55].

### 3.2. Tumour-Infiltrating Lymphocytes

Offering an additional avenue for T cell-based therapeutics, a relatively high neo-antigen load has been observed across a range of primary non-TNBC, and importantly, TNBC samples. Dendritic cell-mediated presentation of these antigens can recruit cytotoxic T lymphocytes (CTLs) [56], highlighting the potential of harnessing patients’ endogenous T cells to treat the disease. This is supported by clinical observations showing that higher levels of tumour-infiltrating lymphocytes (TILs) correlate with more favourable prognoses [57], and multiple trials are currently underway to further stratify TIL levels in relation to the efficacy of neoadjuvant checkpoint inhibitors (NCT03815890, NCT03971045, NCT04307329).

Ongoing clinical investigations are exploring the adoptive transfer of autologous TILs (e.g., NCT04111510, NCT01174121). In this approach, tumour-extracted TILs are expanded ex vivo and reinfused, potentially trading functional exhaustion of tumour-reactive clones for increased absolute numbers [58]. This has been a successful approach in the treatment of metastatic melanoma [59], showing better long-term disease control over ipilimumab leading to the first approval of a TIL therapy for any cancer in February 2024.

### 3.3. Other Approaches (CAR NK, γδ T Cells, TCR-T Cells)

Currently at the preclinical stage for breast cancers, engineered TCRs present a potential therapeutic approach. For example, Kortleve et al. [60] have panned for highly specific TCRs against the breast cancer-associated antigen Rhophilin-associated protein 1 (ROPN1). This antigen is naturally present on testicular cells which lack MHC and hence is not presented to the immune system. Many cancers over-express ROPN1, but being an intracellular protein, it is not amenable to CAR-T therapeutics, but may represent an opportunity for TCR-engineered T cell approaches. Indeed, preclinical testing has demonstrated superior antitumour efficacy in vivo and in 3D tumoroid models of ROPN1-targeting TCR T cells vs. standard of care drugs. One of the major problems with engineered TCR therapeutics is that tumours can downregulate MHC and thus antigen presentation, consequently avoiding this method of targeting [61].

In a similar vein, Janssen et al. [62] demonstrated the preclinical efficacy of engineering conventional αβ T cells with matched γδ TCR chains derived from tumour-resident γδ T cells extracted from breast cancer tumour samples. This approach may enable broader tumour reactivity and reduced off-target toxicity through γδ TCRs while leveraging the enhanced expansion and persistence of conventional αβ T cells. However, the reliance on patient-derived γδ TCRs necessitates individualised sequencing and vector development, potentially limiting scalability due to lengthy production workflows.

Repurposing another innate immune cell type, CAR-NK cells may also hold promise as a breast cancer therapy. NK cells are primed to attack and eliminate cells expressing stress signals or lacking MHC expression, but not MHC presented antigens. This reduces the risk of graft-versus-host disease and enables the development of off-the-shelf cellular products. Cord blood-derived NK cells can also be expanded and deliver enough products for multiple patients [63]. However, NK cells lack the persistence and proliferative capacity of conventional T cells, which may limit long-term tumour control.

As summarised by Adrea et al. [64], over 70 clinical trials are currently registered that are investigating CAR-NK cells for haematological malignancies, where promising efficacy and favourable safety profiles have been reported [65,66,67]. To date, however, no clinical data have been published on the safety or efficacy of CAR-NK therapy in solid tumours. Nonetheless, several trials are ongoing in a range of solid cancers, including breast cancer. These include CAR-NK cells targeting NKG2D ligands (NCT05528341) and Claudin-6, GPC3, mesothelin, or AXL (NCT05410717), with the former utilising irradiated NK-92 cells as an ‘off-the-shelf’ product rather than primary or iPSC-derived NK cells.

## 4. Challenges and Future Direction

Over the past decade, advances in our understanding of breast cancer biology, paired with breakthroughs in immunotherapy, have translated into remarkable improvements in overall survival, offering patients more effective and personalised treatment options. Much of this success stems from the integration of immunotherapies that harness the intrinsic ability of T cells to recognise and destroy cancer cells. Checkpoint inhibitors, for instance, have helped reinvigorate exhausted T cells and restore antitumour immunity in selected breast cancer subtypes. Parallel to this, deeper insights into the molecular pathways that drive breast cancer progression have enabled the development of targeted therapies that disrupt signalling networks essential for tumour survival. This tells us that in principle immunotherapy does work, but the next leap requires a targeting approach, powerful enough to cure breast cancer by overcoming the resistance mechanisms which pose a challenge for current ICIs. Overactivation of the immune system can result in significant toxicities, often occurring before patients derive any therapeutic benefit from ICIs. Another major barrier to their efficacy is the tumour microenvironment (TME). In collagen-rich tumours, ICIs frequently accumulate at the tumour periphery, leaving the majority of tumour cells largely inaccessible. Additionally, regulatory T cells recruited by the tumour suppress the function of activated T cells, while myelosuppressive macrophages further dampen antitumor immune responses [68].

CAR-T cell therapy may represent this next frontier that could overcome those challenges. Unlike conventional immunotherapies, CAR-T cells bypass MHC-dependent antigen presentation and are engineered to recognise tumour antigens directly, allowing for robust and selective tumour killing in a largely antigen-agnostic manner. However, translating the success of CAR-T cells from haematological malignancies into solid tumours such as breast cancer has so far proven challenging. One of the major barriers is the limited expansion and persistence of CAR-T cells in solid tumour environments, particularly when compared to their kinetics when targeting blood cancers. The breast tumour microenvironment is highly immunosuppressive, characterised by inhibitory cytokines, physical barriers, and metabolically hostile conditions, which can collectively blunt the activity of adoptively transferred T cells. However, the most important bottleneck is possibly the identification of true cancer neo-antigens that can be found on the surface of malignant cells without significant expression on normal tissues. So far, CAR-T cells have shown some efficacy in paediatric neuroblastoma [69] and colorectal cancer [70]. In many other tumours, although they have induced a response and were promising early phase trials, the responses were weak. Exhaustion of CAR-T cells and lack of sufficient expansion led to poor persistence and eventually tumour relapse.

These challenges, however, do not represent an insurmountable hurdle, and while the field is still in its infancy, innovative solutions are rapidly emerging. Armoured CAR-T cells, engineered to resist or remodel the tumour microenvironment, are already in development. These next-generation designs include CAR-T cells capable of secreting supportive cytokines such as Il-18, resisting inhibitory signals, or degrading components of the extracellular matrix. Other approaches involve equipping CAR-T cells with therapeutic payload to enhance immune recruitment or directly weaken tumour defences. Gating strategies can also be utilised here to ensure the safety and specificity of CAR-T cell treatments against cancer neo-antigens with off-tumour expression. Combination strategies also hold promise. Radiotherapy, for instance, can increase antigen release and upregulate stress ligands, thereby sensitising tumours to CAR-T-mediated killing. Similarly, monoclonal antibodies, checkpoint inhibitors, and targeted therapies may synergise with CAR-Ts by modulating immune pathways or altering tumour phenotypes [71].

As an alternative to CAR-T therapy, TIL and TCR-engineered T cell therapies have a slightly different targeting approach. By exploiting the natural ability of T cells to recognise peptide–MHC complexes derived from intracellular proteins—an avenue inaccessible to traditional CAR-T designs—they may turbo-boost T cell-based responses against breast cancer. In parallel, emerging research suggests that CAR-T cells directed against cancer-associated stress ligands or conserved “induced self” antigens that are aberrantly expressed in cancer may lead to the development of broad, near-universal CAR-T platforms capable of attacking malignant cells across multiple cancer types.

## 5. Conclusions

The field of breast cancer therapy has evolved massively in the last decade. CAR-T cell therapy may represent the next frontier that could lead to a paradigm shift in how relapsed or refractory disease is treated. In parallel, advances in checkpoint blockade, vaccine development, and combination regimens targeting overactive or overexpressed signalling pathways in breast cancer further expand the therapeutic landscape. Ultimately, the most effective future strategies will likely integrate multiple modalities—engineering, immunology, and precision oncology—to create durable, personalised, and broadly accessible treatments for patients with breast cancer.

## Figures and Tables

**Table 1:** Key phase III trials with immune checkpoint inhibitors for breast cancer.

Trial Name (NCT #)	Phase	Regimen	Target Population	Status
KEYNOTE-522 (NCT03036488)	III	Pembrolizumab + chemo (neoadjuvant) → adjuvant pembrolizumab	Early-stage TNBC	Positive. Approved (FDA).
Impassion 031 (NCT03197935)	III	Atezolizumab + chemotherapy (neoadjuvant)	Early-stage TNBC	Completed. Positive.
KEYNOTE-756 (NCT03725059)	III	Neoadjuvant chemotherapy +/− pembrolizumab → adjuvant pembrolizumab + endocrine therapy	High-risk, ER+/HER2− early breast cancer	Active, not recruiting.
CheckMate 7FL (NCT04109066)	III	Neoadjuvant chemotherapy +/− nivolumab → adjuvant nivolumab + endocrine therapy	High-risk, ER+/HER2− early breast cancer	Active, not recruiting.
NCT02954874	III	Adjuvant pembrolizumab	Early-stage TNBC with residual disease after neoadjuvant chemotherapy	Active, not recruiting. Awaiting final results.
KEYNOTE-355 (NCT02819518)	III	Pembrolizumab + chemo (nab-paclitaxel, gemcitabine/carboplatin)	Metastatic TNBC (PD-L1 CPS ≥ 10)	Completed. Approved (FDA)
NEOTrip (NCT02620280)	III	Atezolizumab + carboplatin/nab-paclitaxel	Early-stage TNBC	Completed—awaiting results.
TROPION-Breast03 (NCT05629585)	III	Dato-DXd +/− durvalumab	Early-stage TNBC without pCR following neoadjuvant therapy	Active—not recruiting

Symbol: “#”: Number; “→” followed by; “+/−”: with or without.

**Table 2:** CAR targets by subtype.

Subtype	Top Targets	Alternative Targets	Key Challenges
HER2+	HER2, B7-H3, MUC1	CLDN6, EGFR	On-target toxicity (HER2).
HR+/HER2−	HER2-low, FRα, CD70	CD44v6, PTK7	Immunosuppressive TME.
TNBC	B7-H3, ROR1, TROP2	NKG2D ligands, CD70	Antigen heterogeneity.
IBC/Metastatic	HER2, EGFR, B7-H3	CD133, CD44v6	Stromal barriers, immune exclusion.

## Data Availability

There are no relevant data associated with this article.
